# Nicotinamide and Pyridoxine in Muscle Aging: Nutritional Regulation of Redox, Inflammation, and Regeneration

**DOI:** 10.3390/antiox14080911

**Published:** 2025-07-25

**Authors:** Agnieszka Nowacka, Maciej Śniegocki, Martyna Śniegocka, Ewa A. Ziółkowska

**Affiliations:** 1 Department of Neurosurgery, Collegium Medicum in Bydgoszcz, Nicolas Copernicus University in Toruń, ul. Curie Skłodowskiej 9, 85-094 Bydgoszcz, Poland; a.nowacka@cm.umk.pl (A.N.); sniegocki@cm.umk.pl (M.Ś.); 2 Department of Anatomical, Histological, Forensic & Orthopedic Sciences, Section of Histology & Medical Embryology, Sapienza University of Rome, Via A. Scarpa, 14-16, 00161 Rome, Italy; martyna.sniegocka@gmail.com; 3 Department of Pediatrics, School of Medicine, Washington University in St. Louis, St. Louis, MO 63110, USA

**Keywords:** nicotinamide, pyridoxine, muscle regeneration, sarcopenia, nicotinamide adenine dinucleotide (NAD^+^) metabolism, muscle stem cells, inflammaging, nutritional intervention

## Abstract

Sarcopenia, the progressive loss of muscle mass, strength, and regenerative capacity with age, is driven by interconnected processes such as oxidative stress, chronic inflammation, mitochondrial dysfunction, and reduced activity of muscle stem cells. As the population ages, nutritional strategies that target these mechanisms are becoming increasingly important. This review focuses on nicotinamide (vitamin B3) and pyridoxine (vitamin B6), two essential micronutrients found in functional foods, which play complementary roles in redox regulation, immune balance, and muscle repair. Nicotinamide supports nicotinamide adenine dinucleotide (NAD^+^) metabolism, boosts mitochondrial function, and activates sirtuin pathways involved in autophagy and stem cell maintenance. Pyridoxine, via its active form pyridoxal 5′-phosphate (PLP), is key to amino acid metabolism, antioxidant defense, and the regulation of inflammatory cytokines. We summarize how these vitamins influence major molecular pathways such as Sirtuin1 (SIRT1), protein kinase B (AKT)/mechanistic target of rapamycin (mTOR), Nuclear factor-κB (NF-κB), and Nrf2, contributing to improved myogenic differentiation and protection of the aging muscle environment. We also highlight emerging preclinical and clinical data, including studies suggesting possible synergy between B3 and B6. Finally, we discuss how biomarkers such as PLP, nicotinamide mononucleotide (NMN), and C-reactive protein (CRP) may support the development of personalized nutrition strategies using these vitamins. Safe, accessible, and mechanistically grounded, nicotinamide and pyridoxine offer promising tools for sarcopenia prevention and healthy aging.

## 1. Introduction

**Figure 1:**
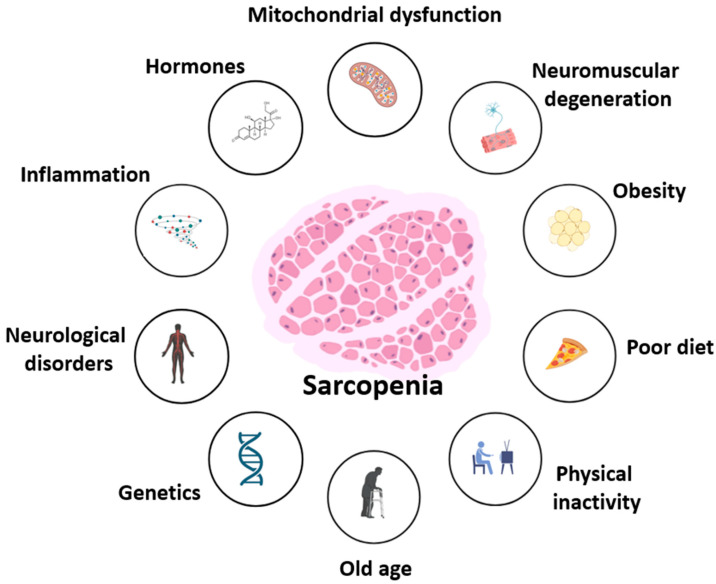
Multifactorial contributors to sarcopenia. Sarcopenia results from interconnected molecular and systemic factors, including mitochondrial dysfunction, hormonal dysregulation, chronic inflammation, neuromuscular and neurological impairment, as well as lifestyle-related contributors such as poor diet, obesity, and physical inactivity. Age-related decline and genetic predisposition further exacerbate muscle loss and functional impairment.

Sarcopenia, the age-related decline in muscle mass, strength, and function, is a critical contributor to frailty, reduced mobility, and loss of independence in older adults [1,2,3]. The pathogenesis of sarcopenia is complex and multifactorial, involving oxidative stress, chronic low-grade inflammation (“inflammaging”), hormonal dysregulation, neuromuscular junction degradation, and impaired satellite cell function (Figure 1). The decline in regenerative potential of skeletal muscle is further compounded by mitochondrial dysfunction, increased extracellular matrix stiffness, and maladaptive immune responses [4,5,6,7,8,9]. As global life expectancy rises, sarcopenia poses an escalating burden on public health systems [6,7,10]. While resistance exercise and adequate protein intake remain the cornerstone of prevention, these strategies may be insufficient for many older individuals due to metabolic resistance or comorbidities. Therefore, functional nutrients that target the molecular drivers of sarcopenia are of increasing interest [10,11,12,13,14,15,16,17,18]. Among them, nicotinamide (vitamin B3) and pyridoxine (vitamin B6) have emerged as promising candidates due to their regulatory roles in redox balance, energy metabolism, inflammatory control, and muscle stem cell activity [19,20,21,22]. Nicotinamide is a precursor of nicotinamide adenine dinucleotide (NAD^+^), a cofactor required for mitochondrial function, sirtuin activity, and DNA repair [20,23,24]. Pyridoxine is a coenzyme in amino acid metabolism, neurotransmitter synthesis, and glutathione production [25,26,27]. Both vitamins are involved in pathways relevant to sarcopenia pathophysiology, including Nuclear factor-κB (NF-κB) signaling, Nrf2 antioxidant responses, and protein kinase B (AKT)/ mechanistic target of rapamycin (mTOR)-mediated protein turnover [28,29,30,31]. Despite their clinical relevance, the therapeutic potential of B3 and B6 in muscle aging remains underexplored in an integrated, mechanistic framework. This review addresses this gap by providing the first comprehensive synthesis of nicotinamide and pyridoxine as dual nutritional modulators of muscle regeneration in aging. We emphasize their converging actions on NAD^+^ metabolism, redox signaling, and immune modulation, highlighting synergistic effects and translational relevance for biomarker-guided, personalized nutrition strategies in sarcopenia prevention.

## 2. Mechanistic Foundations of Skeletal Muscle Aging

### 2.1. Structural and Cellular Organization of Skeletal Muscle

**Figure 2:**
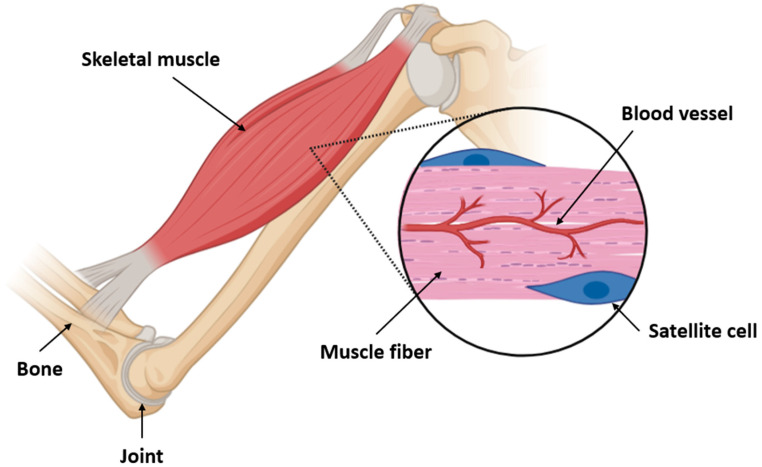
Structural organization of skeletal muscle and localization of satellite cells. This diagram illustrates the anatomical relationship between bones, joints, and skeletal muscles, emphasizing the microarchitecture of muscle fibers. Within the enlarged view, muscle fibers are shown alongside capillary networks (blood vessels) and quiescent satellite cells situated between the basal lamina and sarcolemma. Satellite cells are essential for muscle regeneration and respond to injury or metabolic stress by activating, proliferating, and differentiating into myogenic progenitors. This close spatial association with blood vessels ensures access to nutrients, oxygen, and signaling molecules, critical for regenerative responses in both physiological and pathological conditions such as aging and sarcopenia.

Skeletal muscle is a highly organized and adaptable tissue composed of multinucleated myofibers, connective tissue, blood vessels, and resident stem and immune cells (Figure 2). Each myofiber is encased in a basal lamina and surrounded by a specialized niche containing satellite cells—the adult muscle stem cells that orchestrate repair and regeneration [18,32,33,34,35,36]. These quiescent cells reside beneath the basal lamina and are activated in response to stress, injury, or exercise. Satellite cells are characterized by the expression of transcription factor Pax7 (paired box protein 7) and, upon activation, initiate a myogenic program involving *myogenic factor 5* (*Myf5*) and *myogenic differentiation 1* (*MyoD*) [18,36,37,38,39,40,41]. They proliferate, differentiate into myoblasts, and ultimately fuse with existing fibers or form new fibers to restore tissue integrity. A subset of activated satellite cells self-renews to maintain the stem cell pool. This process is tightly regulated by both intrinsic factors (e.g., epigenetic status and cell polarity) and extrinsic cues from the extracellular matrix (ECM), immune cells, and systemic factors such as hormones and cytokines [18,36,37,38,39,40,41].

### 2.2. Mechanisms of Muscle Aging and Regeneration Decline

**Figure 3:**
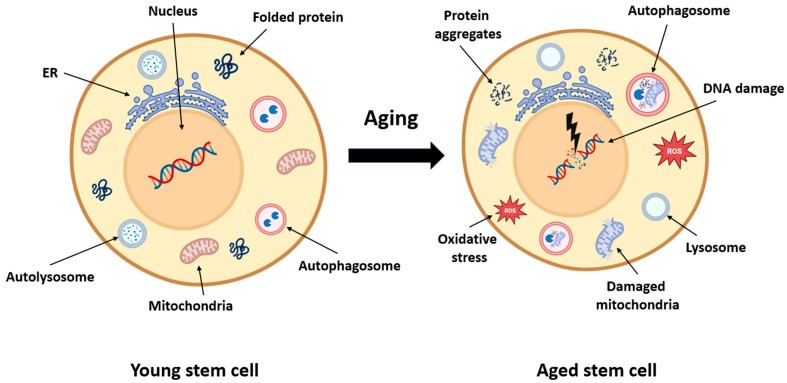
Cellular consequences of aging in muscle stem cells. This schematic compares a young muscle stem cell with an aged stem cell, highlighting the intracellular hallmarks of aging. In youthful cells, mitochondria, protein folding, and autophagy pathways are intact, enabling effective energy production, repair, and proteostasis. With aging, stem cells accumulate protein aggregates, exhibit impaired autophagy, and suffer mitochondrial damage. These changes lead to oxidative stress (↑ Reactive Oxygen Species ROS), DNA damage, and reduced regenerative capacity. Dysfunctional lysosomes and endoplasmic reticulum contribute to further metabolic decline. Collectively, these disruptions impair satellite cell homeostasis, contributing to sarcopenia and diminished muscle repair.

With aging, satellite cell function declines due to both intrinsic changes and an altered niche environment. Aged satellite cells (Figure 3) exhibit diminished proliferative and regenerative capacity, impaired mitochondrial dynamics, reduced autophagy, and increased expression of senescence markers such as p16INK4a, p21, and SA-β-gal [10,18,37,38,42,43,44,45,46].

Telomere shortening and epigenetic drift also contribute to impaired function and replicative exhaustion. Systemically, chronic low-grade inflammation, hormonal imbalance (e.g., reduced insulin-like growth factor 1 (IGF-1) and testosterone), and disrupted nutrient-sensing pathways (e.g., reduced NAD^+^/sirtuin1 (SIRT1) activity and increased mTORC1 hyperactivation) accelerate satellite cell aging [18,38]. The aged ECM becomes stiffer, enriched in crosslinked collagen and fibronectin, and less conducive to regenerative signaling. Increased secretion of TGF-β and fibrogenic cytokines by fibro-adipogenic progenitors (FAPs) promotes fibrosis and impairs satellite cell activation [41,45,47,48,49,50,51,52,53,54,55,56,57,58]. Myofiber atrophy is driven by multiple converging factors: mitochondrial dysfunction leads to ATP depletion and excess reactive oxygen species (ROS) production, NAD^+^ depletion reduces sirtuin activity and impairs mitochondrial quality control, and inflammaging maintains persistent activation of NF-κB, promoting proteolysis via the ubiquitin–proteasome and autophagy–lysosome systems. These disruptions alter signaling through mTOR, adenosine monophosphate-activated protein kinase (AMPK), and Forkhead box O (FoxO) signaling pathways, reducing protein synthesis, increasing degradation, and compromising stress resilience [18,59,60,61,62,63,64,65,66,67,68]. Progressive loss of neuromuscular junction (NMJ) integrity and reduced innervation further accelerate sarcopenia. Emerging markers of aging muscle include elevated levels of pro-inflammatory cytokines (e.g., IL-6 and TNF-α), circulating senescence-associated secretory phenotype (SASP) factors, altered mitochondrial DNA content, and increased oxidative damage markers such as 8-oxo-dG and lipid peroxidation products. These biomarkers can inform the early detection of regenerative decline and guide targeted interventions [18,31,38,62,69,70,71,72,73,74,75,76,77,78].

### 2.3. Key Regulators of Regeneration and Their Age-Related Decline

Effective muscle regeneration requires coordinated activity of several pathways:

- Wnt and Notch signaling regulate satellite cell fate decisions.
- Hypoxia-inducible factor 1-alpha (HIF-1α) and Vascular Endothelial Growth Factor (VEGF) control hypoxia adaptation and angiogenesis.
- mTOR and AKT promote anabolic signaling and protein synthesis.
- SIRT1, an NAD^+^-dependent deacetylase, modulates mitochondrial biogenesis, inflammation, and autophagy.

In aging, dysregulation of these networks contributes to impaired regeneration. For example, chronic activation of mTORC1 in aged muscle promotes anabolic resistance, while declining SIRT1 activity exacerbates oxidative stress and inflammation [7,18,38,54,75,79,80,81,82,83,84,85,86,87].

### 2.4. Prerequisites for Muscle Repair

Effective muscle regeneration requires a temporally coordinated sequence of events. Initially, sterile inflammation following injury activates resident immune cells such as neutrophils and monocytes, which differentiate into pro-inflammatory (M1) macrophages [18,41,54,85,88,89,90]. These macrophages clear debris and secrete cytokines like TNF-α and IL-6 to activate satellite cells. In later stages, M2-like macrophages promote tissue repair by releasing anti-inflammatory cytokines (e.g., IL-10 and TGF-β) and growth factors such as IGF-1 and Hepatocyte Growth Factor (HGF) [38,91,92,93,94,95,96]. Satellite cell activation is followed by proliferation, differentiation, and fusion into multinucleated myofibers. This process is tightly linked to matrix remodeling mediated by fibroblasts and fibro-adipogenic progenitors (FAPs). FAPs support satellite cell function via secretion of Wnt1 and IL-33 but can also drive fibrosis under dysregulated conditions. ECM remodeling involves the balanced activity of matrix metalloproteinases (MMPs) and their inhibitors (TIMPs), facilitating proper scaffold reconstruction [18,38,41,54,85,97,98,99,100]. Nutrient and oxygen supply must be sufficient to support anabolic metabolism and biosynthetic demands. Angiogenesis, mediated by VEGF and angiopoietin signaling, ensures adequate perfusion. Micronutrients like nicotinamide and pyridoxine contribute by modulating redox status, supporting NAD^+^-dependent enzymatic reactions, and influencing inflammatory resolution and myogenic differentiation [31,95,101,102,103,104,105]. Muscle regeneration is also influenced by neuromuscular and hormonal input. Reinnervation of regenerating fibers and restoration of NMJ function are critical for functional recovery. Endocrine factors such as insulin, IGF-1, and sex steroids enhance satellite cell activation and anabolism. Altogether, successful muscle repair is a highly coordinated process involving immune cell transitions, stem cell dynamics, ECM remodeling, angiogenesis, and metabolic support. Aging disrupts these components at multiple levels, leading to inefficient repair, fibrosis, and progressive decline in muscle quality. Interventions targeting redox balance, immune modulation, and stem cell support—such as supplementation with B3 and B6—offer a promising route to restore regenerative capacity [4,7,18,22,25,38,41,44,85,106,107,108].

## 3. Functional Roles of Nicotinamide and Pyridoxine

### 3.1. Biochemical Structure and Classification

Nicotinamide (vitamin B3) and pyridoxine (vitamin B6) are water-soluble B-complex vitamins with essential roles in cellular metabolism and redox balance.

#### 3.1.1. Nicotinamide (Vitamin B3)

Nicotinamide, also known as niacinamide, is one of the two principal forms of vitamin B3 (the other being nicotinic acid). It is structurally composed of a pyridine ring with a carboxamide group at the 3-position. It serves as a precursor to nicotinamide adenine dinucleotide (NAD^+^) and its phosphate form NADP^+^—crucial cofactors in redox reactions, mitochondrial function, DNA repair, and cellular signaling [20,31,105,109,110].

#### 3.1.2. Pyridoxine (Vitamin B6)

Pyridoxine is one of three naturally occurring forms of vitamin B6, along with pyridoxal and pyridoxamine. All three can be converted to the biologically active form, pyridoxal 5′-phosphate (PLP), which functions as a coenzyme in over 140 enzymatic reactions, including those involved in amino acid metabolism, neurotransmitter biosynthesis, and homocysteine regulation. Structurally, pyridoxine contains a hydroxymethyl group at the 4-position of the pyridine ring, distinguishing it from pyridoxal (an aldehyde) and pyridoxamine (an aminomethyl analog) [111,112,113,114,115].

### 3.2. Absorption, Transport, and TISSUE Distribution

#### 3.2.1. Nicotinamide (Vitamin B3)

Vitamin B3, including both nicotinic acid and nicotinamide, is absorbed primarily in the small intestine, with efficiency depending on dosage and chemical form [24,109,116]. At physiological concentrations, absorption occurs through facilitated diffusion via sodium-dependent transporters such as SLC5A8 and SLC22A13, while at pharmacological doses, passive diffusion dominates. After absorption, nicotinamide is distributed systemically via the portal vein and taken up by tissues through equilibrative nucleoside transporters (ENTs) [24,109,116]. Within cells, it enters the NAD^+^ salvage pathway, where it is converted to nicotinamide mononucleotide (NMN) by nicotinamide phosphoribosyltransferase (NAMPT), then to NAD^+^ by NMN adenylyltransferase (NMNAT). This route is essential in skeletal muscle, which has low expression of enzymes required for de novo NAD^+^ biosynthesis from tryptophan and thus depends heavily on the salvage pathway to maintain NAD^+^ levels and mitochondrial health [32,54,85]. Nicotinamide is also converted into NADP^+^ by NAD^+^ kinase, which is necessary for redox reactions involving glutathione and thioredoxin. Tissue-specific NAD^+^ availability depends on the balance between synthesis and consumption by enzymes such as sirtuins, poly(ADP-ribose) polymerases (PARPs), and CD38, all of which are expressed in muscle tissue and increase their activity in response to oxidative stress and inflammation [105,117,118].

#### 3.2.2. Pyridoxine (Vitamin B6)

Pyridoxine and its vitamers—pyridoxal and pyridoxamine—are absorbed in the jejunum mainly through passive diffusion, although recent evidence suggests that carrier-mediated uptake may also occur. Once absorbed, these forms are transported to the liver, where they are phosphorylated by pyridoxal kinase and oxidized by pyridoxamine-phosphate oxidase to generate pyridoxal 5′-phosphate (PLP), the active coenzyme form [112,113,119,120]. PLP binds tightly to albumin in plasma and is delivered to peripheral tissues, including skeletal muscle. Entry into cells likely occurs via facilitated transport followed by intracellular dephosphorylation and rephosphorylation. In skeletal muscle, PLP is involved in over a hundred enzymatic reactions, including those catalyzed by aminotransferases (e.g., alanine aminotransferase - ALT and aspartate aminotransferase - AST), glutamate decarboxylase, and glycogen phosphorylase [54]. These reactions are essential for amino acid turnover, neurotransmitter synthesis, glycogenolysis, and energy homeostasis. PLP’s ability to chelate aldehydes and act as a carbonyl scavenger also supports its antioxidant function, particularly under conditions of muscle inflammation and metabolic stress [121,122].

### 3.3. Metabolic, Redox, and Immune-Modulatory Functions in Muscle Aging

#### 3.3.1. Nicotinamide (Vitamin B3)

**Figure 4:**
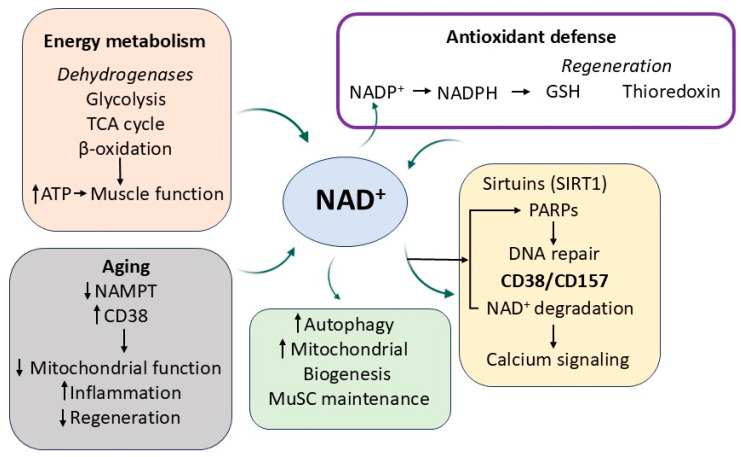
NAD^+^-dependent metabolic and regulatory pathways influenced by nicotinamide in aging skeletal muscle. Nicotinamide supports redox balance, energy metabolism, and gene regulation through its role in NAD^+^ homeostasis. NAD^+^ fuels dehydrogenase activity in key metabolic pathways, supports antioxidant regeneration via NADPH, and regulates sirtuin- and PARP-mediated cellular processes. Age-related NAD^+^ decline, driven by increased CD38 and reduced NAMPT levels, impairs mitochondrial function, increases inflammation, and reduces muscle regeneration.

Nicotinamide plays a central role in redox biology through its involvement in NAD^+^/NADH and NADP^+^/NADPH systems. NAD^+^ is an essential cofactor for dehydrogenase enzymes in glycolysis, the tricarboxylic acid (TCA) cycle, and β-oxidation of fatty acids, supporting energy production in skeletal muscle. NADP^+^, meanwhile, is reduced to NADPH, which acts as a reducing agent in antioxidant defenses, particularly in the regeneration of reduced glutathione (GSH) and thioredoxin. NAD^+^ is also a substrate for several key regulatory enzymes including sirtuins, PARPs, and CD38 [18,31,38,54,74,123,124,125,126,127,128,129,130]. Sirtuins (SIRT1–7) are NAD^+^-dependent deacetylases that modulate gene expression, mitochondrial biogenesis, and oxidative stress responses. In muscle, SIRT1 activation enhances autophagy, protects against oxidative damage, and promotes stem cell maintenance. PARPs are involved in DNA repair, and excessive PARP activity during stress depletes NAD^+^ pools. CD38 and CD157 are NAD^+^-glycohydrolases that regulate calcium signaling and immune responses. CD38 expression increases with age, contributing to systemic NAD^+^ depletion. During aging, NAD^+^ levels decline due to increased consumption by CD38 and decreased activity of NAMPT. This depletion impairs mitochondrial function, disrupts redox homeostasis, and promotes inflammaging, leading to reduced regenerative capacity in muscle stem cells (Figure 4) [18,31,38,54,74,123,124,125,126,127,128,129,130].

Nicotinamide N-methyltransferase (NNMT) is an emerging regulator of NAD^+^ homeostasis, particularly relevant in the context of aging and metabolic dysfunction. NNMT catalyzes the methylation of nicotinamide (NAM) to form 1-methylnicotinamide (MNA), using S-adenosylmethionine (SAM) as the methyl donor. This reaction effectively diverts NAM from the NAD^+^ salvage pathway, reducing the pool of available substrates for NAD^+^ biosynthesis via NAMPT. Elevated NNMT activity has been observed in various pathological conditions, including cancer, obesity, type 2 diabetes, and age-related vascular dysfunction [131,132]. By limiting NAD^+^ availability, NNMT overexpression may suppress the activity of NAD^+^-dependent enzymes such as SIRT1, which plays a key role in autophagy, mitochondrial biogenesis, and anti-inflammatory signaling. Notably, several NNMT inhibitors—including bisubstrate analogs and novel macrocyclic peptides—have been developed and shown to restore intracellular NAD^+^ levels and inhibit MNA production in vitro [133,134,135]. These findings suggest that targeting NNMT may offer a novel strategy to preserve NAD^+^ metabolism, enhance SIRT1 activation, and counteract aging-associated muscle decline.

#### 3.3.2. Pyridoxine (Vitamin B6)

Pyridoxine, in the form of PLP, supports key metabolic processes that influence muscle aging. One-carbon metabolism relies on PLP-dependent enzymes like serine hydroxymethyltransferase (SHMT) and cystathionine β-synthase (CBS), which regulate folate and methionine cycles. These pathways are essential for methylation reactions and homocysteine detoxification [30,54]. Elevated homocysteine levels are associated with increased muscle atrophy, mitochondrial dysfunction, and systemic frailty. In amino acid metabolism, PLP serves as a coenzyme in transamination and decarboxylation reactions that regulate nitrogen balance and neurotransmitter synthesis, including gamma-aminobutyric acid (GABA), serotonin, dopamine, and histamine. PLP-dependent enzymes also modulate branched-chain amino acid (BCAA) metabolism, including leucine, isoleucine, and valine catabolism, which are critical for muscle protein synthesis, nitrogen turnover, and regulation of anabolic signaling pathways like mTOR [136,137]. The glutamine–glutamate cycle, influenced by PLP activity, plays a vital role in nitrogen buffering, antioxidant defense, and excitatory neurotransmission, and is essential for maintaining intracellular redox equilibrium and energy metabolism. Additionally, PLP has antioxidant properties independent of its enzymatic role. It scavenges reactive carbonyl species, including 3-deoxyglucosone, and prevents the formation of advanced glycation end-products (AGEs), which accumulate with aging and contribute to tissue stiffness and inflammation. PLP may also downregulate inflammatory cytokines through modulation of transcriptional pathways, further preserving the muscle microenvironment [54,62,85,122].

#### 3.3.3. Synergistic Antioxidant and Anti-Inflammatory Effects in Muscle Aging

Both nicotinamide and pyridoxine exert significant antioxidant and anti-inflammatory actions, which are particularly relevant in the context of skeletal muscle aging. Nicotinamide enhances SIRT1 activity, resulting in the deacetylation of NF-κB and subsequent repression of pro-inflammatory cytokine expression, including IL-6, TNF-α, and IL-1β [22,24,25,32]. NAD^+^ availability further augments mitochondrial function and improves resistance to reactive oxygen species (ROS)-induced damage by supporting redox cycling and sirtuin-mediated stress responses. Preclinical models have demonstrated that supplementation with nicotinamide or its derivative, nicotinamide mononucleotide (NMN), restores muscle mass, enhances mitochondrial oxidative capacity, and improves physical endurance in aged rodents. These improvements are attributed to restored NAD^+^ levels, enhanced autophagy, and suppression of inflammatory and oxidative pathways [42,55,86,106,130,138,139,140,141,142,143,144].

Clinical observations reveal that low vitamin B6 levels correlate with higher circulating concentrations of C-reactive protein (CRP) and interleukins, including IL-6, and are predictive of reduced grip strength and physical performance in older adults. These findings support a role for vitamin B6 status as both a biomarker and a therapeutic target in muscle aging [18,41,59]. While most studies have investigated the effects of nicotinamide and pyridoxine separately, recent preclinical data suggest that their combination may exert additive or even synergistic effects on muscle stem cell activation and regeneration [22]. In particular, Ancel et al. demonstrated that co-administration of nicotinamide and pyridoxine enhanced MuSC proliferation and differentiation through β-catenin and AKT signaling. These findings, although preliminary, highlight a promising avenue for combined micronutrient strategies. However, further research, especially in clinical settings, is needed to confirm the magnitude, mechanisms, and practical applications of such synergy [18,22,24,41,59,145].

Building on the mechanistic overview presented above, the table below (Table 1) summarizes the primary molecular targets and pathways through which nicotinamide and pyridoxine exert their effects in aging skeletal muscle. These include redox regulation, mitochondrial maintenance, cytokine modulation, and satellite cell activation—each linked to critical outcomes in muscle regeneration and sarcopenia prevention.

## 4. Clinical and Preclinical Relevance and Therapeutic Implications

An expanding body of clinical and preclinical research highlights the therapeutic potential of nicotinamide (vitamin B3) and pyridoxine (vitamin B6) in mitigating sarcopenia. Through complementary effects on NAD^+^ metabolism, mitochondrial function, redox balance, and immune regulation, both vitamins offer promising tools for preserving muscle health in aging populations [22,24,146]. In animal models, nicotinamide riboside (NR) supplementation has been shown to replenish declining NAD^+^ pools, enhance mitochondrial respiration, and activate muscle stem cells (MuSCs). In aged mice, these effects translated into improved muscle endurance and reversal of age-related gene expression signatures [138]. Similarly, nicotinamide mononucleotide (NMN) supported mitochondrial biogenesis and myogenic differentiation and improved survival in ischemic cardiac tissue through poly(ADP-ribose) polymerase 1 (PARP1) inhibition and restoration of mitochondrial enzyme function [144,145,146,147,148]. Human studies offer encouraging, though occasionally inconsistent, findings, with some reporting improved muscle strength, metabolic flexibility, and mitochondrial function, while others show minimal or no effect.

These discrepancies may stem from differences in study populations (e.g., age, sex, health status), variations in the form and dose of supplementation, as well as inconsistent duration and outcome measures. Additionally, small sample sizes and lack of biomarker-based stratification may contribute to limited reproducibility and conflicting results across trials [149,150,151,152,153,154,155,156]. A 12-week randomized trial by Martens et al. reported that NR supplementation increased systemic NAD^+^ levels and reduced arterial stiffness in older adults [149]. Recent clinical evidence demonstrates that oral nicotinamide riboside (NR) supplementation is bioavailable in aged human skeletal muscle, leading to measurable increases in NAD^+^ metabolites and nicotinamide clearance products [150]. Although NR did not significantly alter mitochondrial bioenergetics over the short intervention period, transcriptomic profiling revealed a downregulation of energy metabolism and mitochondrial gene pathways. Importantly, NR supplementation was associated with reduced levels of circulating inflammatory cytokines, suggesting potential anti-inflammatory effects. These findings support the translational relevance of NR for modulating the muscle NAD^+^ metabolome and systemic inflammation in older adults, even in the absence of immediate changes in mitochondrial function [150]. In a 5-month randomized trial involving BMI-discordant monozygotic twins, escalating doses of nicotinamide riboside (250–1000 mg/day) enhanced muscle mitochondrial biogenesis, satellite cell differentiation, and gut microbiota diversity and modulated DNA methylation in muscle and adipose tissue. However, NR supplementation did not lead to improvements in adiposity or overall metabolic health [151]. In a randomized crossover trial, six weeks of nicotinamide riboside (1000 mg/day) in overweight and obese individuals increased skeletal muscle NAD^+^ metabolites and acetylcarnitine concentrations and modestly improved fat-free mass and sleeping metabolic rate. However, no significant effects were observed on insulin sensitivity, mitochondrial function, cardiac performance, or inflammatory markers [152]. Recent preclinical work by Ancel et al. identified a synergistic effect of nicotinamide and pyridoxine on muscle stem cell (MuSC) activation and regeneration. The combination of these food-derived nutrients enhanced MuSC proliferation and differentiation through CK1-dependent β-catenin activation and AKT signaling. In aged mice, oral administration of NAM and PN significantly improved muscle strength and regeneration following injury. Furthermore, their levels declined with age and were positively associated with muscle mass and walking speed in a cohort of 186 elderly individuals, highlighting their clinical relevance and potential as nutritional rejuvenators of MuSC function [22].

These mixed results underscore the need for refined strategies that consider dosage, timing, formulation, and individual variability in aging physiology. Although direct evidence of their synergy in skeletal muscle is still limited, preclinical models demonstrate that nicotinamide enhances mitochondrial biogenesis, autophagy, and satellite cell activation, while pyridoxine modulates amino acid metabolism and inflammatory signaling [115,153,154,155,156]. Further investigation into combined supplementation strategies is warranted to determine their potential interactive effects on muscle strength, regeneration, and function in aging populations. Pyridoxine, particularly in its active form pyridoxal-5′-phosphate (PLP), has been consistently associated with better muscle function in observational studies. Additional evidence suggests that physical exercise may influence vitamin B6 dynamics in both humans and animals, further amplifying its role in muscle physiology [115,153,154,155,156]. Multiple human studies have reported transient increases in plasma PLP levels following endurance exercise. For example, adolescent runners exhibited a significant rise in plasma PLP and total B6 concentrations after 4500-m races [157]. Similar results were observed in trained and untrained women following cycling at 80% VO_2_max for 20 min, where PLP and 4-pyridoxic acid (4-PA) levels rose post-exercise [158,159]. Plasma PLP also increased during prolonged running in men [160] and following graded cycling challenges in healthy adults [161]. Venta et al. confirmed these findings in young male athletes, showing significant post-exercise elevations in PLP after a maximal aerobic test [162], while Deiana et al. observed increased serum PLP and PMP after a half-marathon [163]. In animal models, similar trends have been documented. Rats exposed to forced swimming for varying durations showed time-dependent increases in plasma PLP, muscle B6 vitamers, and liver total B6 content [164]. Chronic endurance training over several weeks also elevated tissue levels of PLP, PMP, and PL in skeletal muscle and liver [165]. Notably, Okada et al. demonstrated that exercise reversed the negative effects of a B6-deficient diet on mitochondrial enzymes and muscle B6 levels [166]. While one study reported no change in plasma PLP after an 8-week swimming protocol, this appears to be an exception rather than the norm [167]. Collectively, these findings indicate that endurance exercise enhances tissue and circulating levels of vitamin B6, potentially improving its functional availability during muscle remodeling and repair. This adaptive redistribution of B6 vitamers may augment the vitamin’s antioxidant and anti-inflammatory capacity, particularly in the context of physical rehabilitation or sarcopenia intervention. Given the interaction between exercise and B6 metabolism, future trials should investigate whether co-administration of B6 with structured physical activity provides additive or synergistic benefits for muscle regeneration in aging populations.

Altogether, nicotinamide and pyridoxine target key pathological features of muscle aging, including mitochondrial decline, stem cell exhaustion, and inflammaging. Their clinical effects appear to be amplified when co-administered with structured exercise, adequate protein intake, and other bioactive nutrients such as vitamin D and leucine. Future trials should prioritize identifying optimal nutrient combinations, delivery formats, and biomarker-guided interventions to maximize their regenerative potential in sarcopenia management. However, it is important to note that many of the cited studies share common limitations, including small sample sizes, short intervention durations, and lack of functional or muscle-specific endpoints. Several preclinical studies were conducted in young or non-aged animals, and some findings were derived from in vitro models that may not fully capture the complexity of muscle aging.

A summary of key preclinical and clinical studies exploring the effects of nicotinamide, pyridoxine, and their combinations on muscle metabolism, stem cell activation, and regeneration, including their main limitations, is presented in Table 2.

Above, we provide a summary of experimental and clinical studies evaluating the impact of vitamin B3 (nicotinamide and its derivatives) and vitamin B6 (pyridoxine and PLP) on mitochondrial function, stem cell activity, muscle regeneration, and exercise-induced metabolic adaptations. The table includes both human and animal models, with the findings supporting their roles in enhancing NAD^+^ metabolism, muscle stem cell (MuSC) function, antioxidant defense, and physical performance.

## 5. Dietary Sources, Supplementation, and Formulation Strategies

### 5.1. Natural Dietary Sources

Nicotinamide (vitamin B3) and pyridoxine (vitamin B6) are widely distributed in both plant-based and animal-based food sources, making them accessible components of the human diet. Nicotinamide and its related form, nicotinic acid, are found in significant amounts in meats (especially liver, poultry, and fish), whole grains, legumes, seeds, and fortified cereals [30,105,168,169,170]. Endogenously, nicotinamide is also synthesized through the tryptophan–kynurenine pathway, though this pathway becomes less efficient with age, increasing the importance of dietary sources. Pyridoxine and its bioactive forms—pyridoxal and pyridoxamine—are prevalent in foods such as bananas, avocados, whole grains, nuts, potatoes, and animal products including poultry, fish, and organ meats [122,171,172,173,174,175]. Among these, pyridoxal 5′-phosphate (PLP), the active coenzyme form of B6, is especially critical for amino acid metabolism and neurotransmitter synthesis. Food processing and preparation significantly affect vitamin content. For example, nicotinamide is relatively stable during cooking, whereas pyridoxine is sensitive to heat and can degrade under prolonged boiling or storage. Bioavailability also varies; nicotinamide from animal sources is typically more readily absorbed compared to that from plant-based sources bound within complex food matrices. Both vitamins have been incorporated into food fortification programs, particularly in countries with high prevalence of deficiencies or limited dietary diversity [30,176,177,178]. In elderly populations, ensuring adequate intake from food alone can be challenging due to reduced appetite, gastrointestinal function, or dietary restrictions—making the case for supplemental forms even stronger. Monitoring dietary intake through food frequency questionnaires, 24 h recalls, or biomarker analysis (e.g., serum PLP or urinary metabolites) provides valuable insights into nutrient adequacy and informs public health strategies. Ensuring consistent and sufficient intake of nicotinamide and pyridoxine through a balanced diet—supported by functional food products when necessary—forms a foundational strategy to maintain muscle metabolic function, counter oxidative stress, and promote healthy aging [54,106,122,139,179,180].

### 5.2. Fortification and Supplementation Strategies

Targeted supplementation of nicotinamide and pyridoxine supports muscle regeneration in aging. B3 precursors such as NR and NMN have been shown in trials and animal models to enhance NAD^+^ levels, mitochondrial health, and physical function. Pyridoxine supplementation restores mitochondrial enzyme activity, reduces levels of inflammatory cytokines, and supports protein metabolism [54,181]. These vitamins are available in B-complex formulas, microencapsulated products, or protein-fortified functional foods. Combining them with nutrients like folate or omega-3s may enhance effects. Both are safe at recommended doses, though excessive B6 can cause neuropathy. In clinical settings, especially among frail or hospitalized older adults, personalized supplementation guided by biomarkers may complement physical therapy and nutrition protocols. These strategies have the potential to be integrated into public health efforts to prevent or mitigate sarcopenia [1,2,10,18,54,181,182,183,184,185,186,187].

## 6. Nutrient Interactions and Personalized Nutrition Strategies

**Figure 5:**
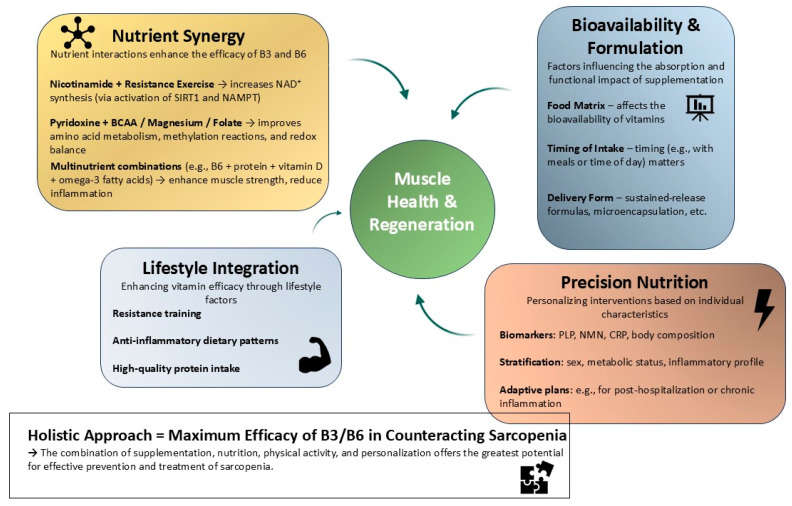
Integrative framework for the role of nicotinamide (B3) and pyridoxine (B6) in skeletal muscle regeneration. This conceptual diagram illustrates a multifactorial strategy through which nicotinamide and pyridoxine support muscle regeneration and help counteract sarcopenia. Nicotinamide interacts synergistically with resistance exercise to enhance NAD^+^ synthesis via the SIRT1–NAMPT pathway, while pyridoxine acts in concert with amino acids and micronutrients to improve redox balance and metabolic regulation. Key factors such as the food matrix, timing of intake, and delivery form influence vitamin uptake and bioefficacy. Precision nutrition approaches, guided by biomarkers (e.g., PLP, NMN, and CRP) and individual phenotypic profiling, enable targeted intervention design. Integration with lifestyle factors such as resistance training, anti-inflammatory dietary patterns, and adequate protein intake further amplifies the regenerative impact. This framework highlights the importance of combining molecular, nutritional, and behavioral components for optimal muscle health in aging.

The biological effects of nicotinamide and pyridoxine do not occur in isolation; rather, they are modulated by their interactions with other nutrients and lifestyle factors. These interactions can amplify their effects on muscle metabolism, stem cell function, and systemic health, especially when incorporated into personalized nutrition strategies [188,189,190]. A growing body of research supports the concept of nutrient synergy in muscle health. For instance, combining nicotinamide with resistance exercise enhances NAD^+^ biosynthesis and mitochondrial adaptations. Nicotinamide supplementation increases SIRT1 activity, while exercise-induced NAMPT expression synergistically boosts NAD^+^ salvage [54,191,192,193]. Similarly, pyridoxine interacts with branched-chain amino acids and micronutrients like magnesium and folate to improve amino acid metabolism, methylation reactions, and redox balance. Multinutrient supplementation trials in frail elderly populations show that B6, when combined with adequate protein, vitamin D, and omega-3 fatty acids, is associated with improved muscle strength, reduced inflammation, and slower lean mass loss [85,155,156,188]. This suggests that co-formulation and dietary pattern design are key to maximizing efficacy. Moreover, the food matrix and timing of intake influence the bioavailability and metabolic fate of both B3 and B6, indicating the importance of holistic nutritional planning. The development of precision nutrition approaches offers new opportunities for tailoring interventions based on individual nutritional phenotypes and risk profiles. Biomarkers such as plasma PLP, urinary NMN, CRP, and body composition indices can guide personalized plans [194,195,196,197]. Stratifying patients by sex, metabolic status, and inflammatory burden may enhance the effectiveness of B3/B6 supplementation. Additionally, elderly individuals with chronic conditions or post-hospitalization recovery may benefit from customized combinations of dietary and lifestyle interventions. Integrating vitamin B3 and B6 supplementation with resistance training, high-quality protein intake, and anti-inflammatory dietary patterns could restore regenerative capacity and improve quality of life. Altogether, nutrient interactions and personalized nutrition represent a promising direction for harnessing the full potential of nicotinamide and pyridoxine in combating sarcopenia. This multifaceted strategy leverages dietary complexity, metabolic individuality, and molecular biology to support muscle health in aging populations [1,2,18,19,22,185] (Figure 5).

## 7. Future Directions

Despite growing interest in nicotinamide and pyridoxine as modulators of muscle aging, several critical gaps remain in our understanding of their mechanisms, optimal applications, and long-term outcomes. Addressing these gaps will require coordinated advances in basic science, clinical research, and precision nutrition. One major challenge is the limited availability of robust, long-term clinical data assessing the effects of B3 and B6 supplementation on sarcopenia-related outcomes such as muscle mass, strength, physical performance, and regenerative capacity. Most existing trials are short in duration, focus on surrogate markers, and often exclude vulnerable populations such as the frail elderly or individuals with multimorbidity. Future randomized controlled trials (RCTs) should prioritize longer follow-up periods, diverse populations, and clinically meaningful endpoints, including biomarkers of NAD^+^ metabolism (e.g., NMN and NAMPT), inflammation (e.g., CRP and IL-6), and muscle regeneration (e.g., circulating myokines and MuSC activity). Additionally, the mechanistic basis for vitamin–exercise–diet interactions needs to be explored in greater detail. For example, how do different forms of vitamin B3 (e.g., NR vs. NMN) modulate muscle adaptation to resistance or endurance training? Does the timing of B6 intake influence amino acid signaling and muscle protein synthesis post-exercise? These questions can be addressed through integrative approaches combining omics technologies, satellite cell tracing, and metabolic flux analysis in animal models and humans. Another underexplored area is the potential of nicotinamide and pyridoxine to modify the muscle stem cell niche and delay the onset of cellular senescence. Investigating whether these vitamins impact SASP (senescence-associated secretory phenotype), epigenetic remodeling, or stem cell exhaustion could unveil new therapeutic opportunities. This is especially relevant in the context of inflammaging, where low-grade inflammation perpetuates tissue damage and impairs regeneration. From a translational perspective, the development of precision nutrition tools—including wearable sensors, machine learning algorithms, and digital dietary assessments—may enable more accurate tracking of nutrient status and facilitate individualized supplementation plans. Integration with molecular biomarkers could help identify responders vs. non-responders and refine dosing regimens based on metabolic and genetic profiles. Finally, public health strategies should consider how nicotinamide and pyridoxine can be incorporated into aging-focused dietary guidelines, functional food innovation, and rehabilitation protocols. Education campaigns targeting older adults, caregivers, and clinicians could promote safe and effective use of these vitamins as part of comprehensive sarcopenia prevention programs.

## 8. Conclusions

Sarcopenia is a multifactorial and progressive condition driven by mitochondrial dysfunction, oxidative stress, chronic inflammation, and impaired satellite cell dynamics. As global populations age, addressing these underlying drivers of muscle decline is essential to preserve mobility, independence, and quality of life. This review highlights the complementary and potentially synergistic roles of nicotinamide (vitamin B3) and pyridoxine (vitamin B6) in modulating key molecular pathways involved in skeletal muscle maintenance and regeneration. Nicotinamide supports NAD^+^ metabolism, redox balance, and sirtuin signaling, while pyridoxine contributes to amino acid metabolism, neurotransmitter synthesis, and antioxidant defenses. Both vitamins influence critical regulatory axes, including SIRT1, AKT/mTOR, and NF-κB, with evidence supporting their role in reducing inflammaging and supporting myogenesis. Despite promising findings from preclinical and early clinical studies, several challenges remain. Many trials are short-term, underpowered, or lack functional endpoints. The optimal dosage, duration, and safety of long-term supplementation, especially in older populations, are still unclear. Furthermore, variability in study design and participant characteristics limits direct comparisons. Future research should focus on integrating B3 and B6 into multimodal strategies, including resistance training, adequate protein intake, and vitamin D supplementation, and developing biomarker-guided, personalized interventions. Novel targets such as NNMT inhibition may further enhance NAD^+^ availability and SIRT1 activation. Altogether, nicotinamide and pyridoxine represent accessible, evidence-supported tools with potential to be incorporated into comprehensive approaches for sarcopenia prevention and healthy aging.

## Figures and Tables

**Table 1:** Molecular mechanisms of action of nicotinamide and pyridoxine in aging skeletal muscle.

Mechanism/Pathway	Nicotinamide (B3)	Pyridoxine (B6)	Functional Outcome	Reference
NAD^+^ metabolism	Precursor for NAD^+^, essential for mitochondrial function	Indirect support via amino acid catabolism	Maintains energy homeostasis and redox balance	[18,54,74,123,124,125,126,127,128,129,130]
Sirtuin signaling (SIRT1)	Activates SIRT1 and promotes autophagy and mitochondrial biogenesis	Not directly involved	Enhances mitochondrial health and stem cell longevity	[22,24,25,32,54,106,130]
Redox regulation/Nrf2 pathway	Upregulates antioxidant enzymes via the SIRT1–Nrf2 axis	Scavenges ROS and carbonyl compounds	Reduces oxidative damage	[22,24,25,54,62,85]
Inflammatory signaling (NF-κB)	Inhibits NF-κB and downregulates IL-6, TNF-α	Reduces levels of pro-inflammatory cytokines	Attenuates inflammaging	[22,24,25,32,41,54,106]
Muscle stem cell (MuSC) function	Enhances activation via AKT/mTOR and β-catenin	Supports function via redox control and protein metabolism	Improves regeneration and myogenesis	[18,22,24,41,59,145]

**Table 2:** Clinical and preclinical studies investigating the effects of nicotinamide (vitamin B3) and pyridoxine (vitamin B6) on muscle health and regeneration.

Study Type	Vitamin(s)	Model/Population	Dose	Key Findings	Main Limitations	Reference
Preclinical (mouse)	Nicotinamide (NR)	Aged C57BL/6 mice	Not specified	↑ NAD^+^ levels, ↑ MuSC activation, improved endurance, reversal of aging gene signatures	Limited dosing detail, short-term study	[143]
Preclinical	NMN	Myoblasts/cardiac tissue/ C57BL6/J mice	100 μM (in vitro); 100–500 mg/kg/day (in vivo)	↑ Mitochondrial biogenesis, improved survival via PARP1 inhibition	In vitro relevance, limited muscle-specific outcomes	[144,145,146,147,148]
Clinical (Twin study)	Nicotinamide (NR)	BMI-discordant monozygotic twins (5 mo)	250–1000 mg/day	↑ NAD^+^ metabolism, ↑ mitochondrial biogenesis, ↑ MuSCs, ↑ microbiota diversity, DNA methylation modulation; no metabolic improvement	Small cohort, short intervention	[151]
Clinical (RCT)	Nicotinamide (NR)	Adults (55–79 y/o)	1000 mg/day	↑ Systemic NAD^+^, ↓ arterial stiffness	Short duration	[149]
Clinical (Pilot)	Nicotinamide (NMN)	Healthy older adults (both sexes, ~75 y/o)	1000 mg/day	↑ Muscle NAD^+^ metabolites, altered mitochondrial gene expression, ↓ circulating inflammatory cytokines	Small sample size, no long-term follow-up	[150]
Clinical	Nicotinamide (NR)	Overweight adults	1000 mg/day	↑ NAD^+^ metabolites and acetylcarnitine in muscle, ↑ lean mass, ↑ sleeping metabolic rate; no major metabolic effects	No exercise/nutrition control, non-aging group	[152]
Clinical	Nicotinamide + Pyridoxine (NAM + PN)	Older adults	NAM 1000 mg + PN 200 mg	Enhanced MuSC activation, accelerated regeneration, ↑ strength, ↑ walking speed	Preliminary findings, limited mechanistic data	[22]
Human Exercise Study	Pyridoxine (PLP)	Male adolescent athletes (4500 m run)	Not specified	↑ PLP and total B6 after exercise	Observational, no long-term effects	[157]
Human Exercise Study	Pyridoxine (PLP)	Trained/untrained women (cycling)	8.0–10.4 mg/day	↑ PLP and 4-PA post-exercise	Small sample, unbalanced baseline activity	[158,159]
Human Exercise Study	Pyridoxine (PLP)	Men (2 h run at 60–65% VO_2_max)	Not specified	↑ Plasma PLP during exercise	Single time point, no control group	[160]
Human Exercise Study	Pyridoxine (PLP)	Adults (cycling at 60% and 85% VO_2_max)	Not specified	↑ PLP concentration, peak within 5 min	Short-term response only, small sample	[161]
Human Exercise Study	Pyridoxine (PLP)	Male athletes (VO_2_max exhaustion test)	Not specified	↑ PLP after maximal aerobic exercise	Acute response, limited generalizability	[162]
Human Exercise Study	Pyridoxine (PLP)	Male amateur runners (half-marathon)	Not specified	↑ Serum PLP and PMP post-race	No control group	[163]
Animal Exercise Study	Pyridoxine (PLP)	Rats (acute forced swimming)	Not specified	↑ Plasma/muscle/liver B6 vitamers with increasing swim duration	Short exposure, not aged animals	[164]
Animal Exercise Study	Pyridoxine (PLP)	Rats (9-week swim training)	7 mg/kg/day	↑ Muscle and liver PLP, PMP, and PL; no change in plasma PLP	No histological assessment, non-aged rats	[165]
Animal Exercise Study	Pyridoxine (PLP)	Wistar rats	1.5 mg/kg/day	Reversal of B6 deficiency effects on mitochondrial enzymes	Limited translational value	[166]
Animal Exercise Study	Pyridoxine (PLP)	Sprague Dawley rats	Not specified	No change in plasma PLP	No mechanism explored	[167]

## Data Availability

No new data were created or analyzed in this study. Data sharing is not applicable to this article.
